# Design and Synthesis of Hollow Nanostructures for Electrochemical Water Splitting

**DOI:** 10.1002/advs.202105135

**Published:** 2022-01-18

**Authors:** Min Yang, Cai Hong Zhang, Nian Wu Li, Deyan Luan, Le Yu, Xiong Wen (David) Lou

**Affiliations:** ^1^ State Key Lab of Organic‐Inorganic Composites Beijing University of Chemical Technology Beijing 100029 P. R. China; ^2^ School of Chemical and Biomedical Engineering Nanyang Technological University 62 Nanyang Drive Singapore 637459 Singapore

**Keywords:** architecture optimization, compositional manipulation, hollow nanostructures, water electrolysis

## Abstract

Electrocatalytic water splitting using renewable energy is widely considered as a clean and sustainable way to produce hydrogen as an ideal energy fuel for the future. Electrocatalysts are indispensable elements for large‐scale water electrolysis, which can efficiently accelerate electrochemical reactions occurring at both ends. Benefitting from high specific surface area, well‐defined void space, and tunable chemical compositions, hollow nanostructures can be applied as promising candidates of direct electrocatalysts or supports for loading internal or external electrocatalysts. Herein, some recent progress in the structural design of micro‐/nanostructured hollow materials as advanced electrocatalysts for water splitting is summarized. First, the design principles and corresponding strategies toward highly effective hollow electrocatalysts for oxygen/hydrogen evolution reactions are highlighted. Afterward, an overview of current reports about hollow electrocatalysts with diverse architectural designs and functionalities is given, including direct hollow electrocatalysts with single‐shelled, multi‐shelled, or open features and heterostructured electrocatalysts based on hollow hosts. Finally, some future research directions of hollow electrocatalysts for water splitting are discussed based on personal perspectives.

## Graphical abstract

Hollow nanostructures with diverse architectural features and functionalities have attracted considerable interest as promising electrocatalysts for water splitting. Herein, some recent progress in the design of micro‐/nanostructured hollow structures for electrocatalytic water splitting and beyond is summarized. Special focus is taken on the direct hollow electrocatalysts with single‐shelled, multi‐shelled, or open features and heterostructured electrocatalysts based on hollow hosts.

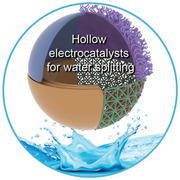

## 1 Introduction

Along with the depletion of conventional fossil fuels and global warming issue because of the increasing carbon dioxide emissions, major concerns about the energy future have triggered the research community to search a clean and sustainable fuel‐combustion technology from fossil‐free pathways.^[^ ^1^, ^2^, ^3^, ^4^, ^5^ ^]^ Particularly, hydrogen has been identified as an ideal alternative energy fuel as its combustion product is only water. Water electrolysis is considered as one of the promising and zero‐carbon‐emitting strategies for hydrogen production compared to the traditional steam reforming or coal gasification based on the reactions between fossil fuel and steam.^[^ ^6^, ^7^, ^8^ ^]^ However, it remains challenging to achieve highly efficient production of hydrogen at industrial scale using renewable energy. A crucial step for realizing this goal is the development of cost‐effective electrocatalysts to accelerate the sluggish kinetics of hydrogen evolution reaction (HER) at the cathode and oxygen evolution reaction (OER) at the anode.^[^ ^9^, ^10^, ^11^ ^]^ Currently, noble metals and their compounds are state‐of‐the‐art electrocatalysts for water‐splitting reactions at both ends (e.g., Pt for HER and IrO_2_ or RuO_2_ for OER).^[^ ^12^, ^13^, ^14^ ^]^ Nevertheless, the widespread use of these electrocatalysts still suffers from scarce resource, prohibitive cost of noble metal, and lack of long‐term stability.^[^ ^15^, ^16^, ^17^, ^18^ ^]^

Enormous efforts have been made to rationalize electrocatalyst performances at relatively low cost. Generally speaking, the strategies for improving the activity of electrocatalysts can be classified into two types.^[^ ^19^, ^20^, ^21^ ^]^ One is to increase the apparent activity of total electrode via structural regulation. The other type is to improve the intrinsic activity of each active site through compositional engineering. Particularly, hollow nanostructures could simultaneously integrate the above approaches to maximize their benefits.^[^ ^22^, ^23^, ^24^, ^25^, ^26^ ^]^ To be specific, the high surface area of hollow nanostructures provides plenty of accessible active sites. Compared to other nanostructured electrocatalysts with high surface area, the hollow configurations demonstrate strong confinement effects. To be specific, hollow‐structured electrocatalysts with porous shells show significant improvement in protecting particles from migration and aggregation.^[^ ^21^, ^25^ ^]^ More importantly, tunable mass transport during reactions could be achieved by regulating the shell structure.^[^ ^27^, ^28^ ^]^ Tunable chemical compositions of hollow materials enable the optimization of binding and desorption of reaction intermediates.^[^ ^29^ ^]^ Moreover, hollow nanostructures can largely reduce the usage of expensive noble metals. According to the function in water splitting, hollow nanostructures could serve as direct electrocatalysts or hosts for loading active materials.^[^ ^22^, ^25^, ^30^ ^]^ For the former type, hollow materials are the direct active species to catalyze water splitting at their surface. As for the latter one, active components are loaded onto the pores/channels, outer/inner surfaces, interior cavities or encapsulated in the frame of hollow hosts to form heterostructured electrocatalysts. Hollow materials are employed as the conductive network, surface modifier, or structural stabilizer for enhancing the performances of active species.

Depending on the reactions in water splitting and functions, design principles of advanced hollow electrocatalysts are quite distinctive. The compositional manipulation for HER and OER catalysts is based on the binding energies of electrocatalyst surface with reactive intermediates.^[^ ^1^, ^31^, ^32^ ^]^ Usually, material selections for these two reactions are not the same. The atomic‐level descriptor of two‐electron HER is hydrogen binding energy over the full range of pH conditions.^[^ ^33^, ^34^, ^35^, ^36^ ^]^ Whereas, the electrocatalytic activities of OER electrocatalysts depend largely on the stabilities of adsorbed intermediates (OH*, O*, OOH*) on the surface of catalysts and evaluated by the descriptor of free energy difference, i.e., Δ*G*_O*_ – Δ*G*_OH*_.^[^ ^37^, ^38^, ^39^ ^]^ In addition, the architecture optimization criterions for hollow structured electrocatalysts and support materials are quite different. As for direct catalysts, maximization of exposed electrocatalytic active sites is the priority. Therefore, structural evolution of building blocks from 0D nanoparticles to 1D or 2D high‐energy facets plays a vital role to increase the number of reactive sites. Construction of complex hollow nanostructures with multiple shells or complex internal voids could be helpful for direct catalysts to enhance the loading of active species per unit area. Beyond that, the employment of hollow structures with open features could effectively facilitate the charge and mass transfer.^[^ ^40^, ^41^, ^42^ ^]^ As for the host structures, modulation approaches focus on the integration of electroactive components into hollow supports.^[^ ^30^ ^]^ Conductive hollow hosts could not only improve the electronic conductivity of electrocatalysts, but also prevent the particle aggregation during electrocatalytic reactions. Last but not the least, simplification of synthetic route is a crucial issue for the wide application of hollow structured electrocatalysts. Generally speaking, the preparation procedure for the direct electrocatalysts is much more convenient than that for the heterostructured electrocatalysts. For the typical synthesis of hollow structures, hard/soft‐templating methods are usually involved, where the template acts as a physical scaffold for the target material coating and is removed subsequently to form void space.^[^ ^21^ ^]^ Notably, self‐templated strategies are gaining rapidly increasing attention, which could directly convert reactant into hollow structured product without extra template removal process. Based on the formation mechanism of internal voids, self‐templated strategies can be divided into controlled etching, outward diffusion, and heterogenous contraction.^[^ ^43^, ^44^ ^]^ The rapid development of template‐involved strategies from hard/soft templating to self‐templated methods has motivated the evolution and optimization of hollow catalysts.^[^ ^43^, ^45^ ^]^

Several review and perspective articles have critical discussions of the design strategies and syntheses of hollow structured catalysts for various catalytic applications.^[^ ^22^, ^23^, ^24^, ^26^, ^30^, ^46^, ^47^ ^]^ In this perspective, we summarize some recent progress in the development of hollow catalysts for electrocatalytic water splitting. Special emphasis will be placed on the structural/compositional evolution of hollow nanostructures as direct electrocatalysts and hosts. Particularly, the architectures for direct catalysts are categorized into hollow materials with single‐shelled, multi‐shelled, and open features. Host materials are categorized into heterostructured electrocatalysts loaded on hollow hosts and integrated hollow nanoreactor. In the final part, we provide a brief discussion about both the challenges and directions toward the future design of hollow electrocatalysts for water electrolysis.

## 2 Hollow Direct Electrocatalysts for Water Splitting

**Figure 1:**
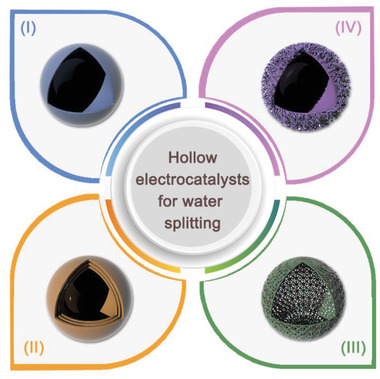
Schematic illustration of advanced hollow structured electrocatalysts for water splitting: I) single‐shelled hollow catalysts, II) multi‐shelled hollow catalysts, III) heterostructured catalysts loaded on hollow hosts, and IV) integrated hollow nanoreactor.

As illustrated in **Figure** **1**, the hollow catalysts for electrochemical water splitting can be classified into direct electrocatalysts and heterostructured electrocatalysts composed of catalysts and hosts. According to the geometrical and compositional features, direct electrocatalysts can be divided into hollow structures with single‐shelled, multi‐shelled, or frame‐like open features, whereas heterostructured electrocatalysts can be electrocatalyst loaded on hollow hosts or integrated hollow nanoreactors, where electrocatalysts and hosts cannot be clearly identified. In the following sections, we highlight some representatives of these novel hollow electrocatalysts for water splitting to elucidate the underlying structure–activity relationship and design principle from simple to complex.

### 2.1 Single‐Shelled Hollow Structures as Direct Electrocatalysts

#### 2.1.1 Simple Hollow Electrocatalysts

**Figure 2:**
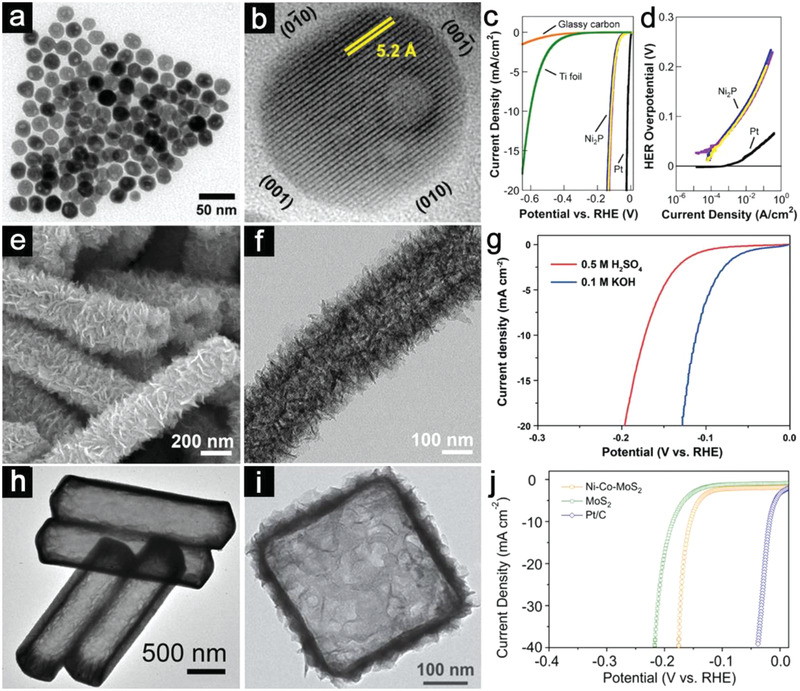
a) TEM image of Ni_2_P nanoparticles. b) HRTEM image of a single Ni_2_P hollow particle. c) Polarization curves of three Ni_2_P electrodes and comparative groups. d) Tafel plots for the Ni_2_P and Pt electrodes. (a–d) Reproduced with permission.^[^ ^49^ ^]^ Copyright 2013, American Chemical Society. e) FESEM image of *β*‐Mo_2_C nanotubes. f) TEM image of an individual *β*‐Mo_2_C nanotube. g) Tafel plots for the *β*‐Mo_2_C nanotubes in acid and alkaline solutions. (e–g) Reproduced with permission.^[^ ^50^ ^]^ Copyright 2015, Wiley‐VCH. h) TEM image of CoMoS_4_ hollow prisms. Reproduced with permission.^[^ ^51^ ^]^ Copyright 2016, John Wiley and Sons. i) TEM image of an individual Ni‐Co‐MoS_2_ nanobox. j) Polarization curves of Ni‐Co‐MoS_2_ nanobox electrode and control samples. Reproduced with permission.^[^ ^52^ ^]^ Copyright 2016, Wiley‐VCH.

Single‐shelled hollow structures are the most common form of direct electrocatalysts for water splitting. Benefitting from the facile preparation and modulation, single‐shelled hollow structures have attracted numerous research attention for various applications since the pioneering work done by Caruso's group in the late 1990s.^[^ ^48^ ^]^ The employment of hollow structures in electrocatalytic hydrogen production could effectively improve the apparent activity of catalysts and reduce the usage of noble Pt metal. As a typical example, nickel phosphide (Ni_2_P) hollow spheres synthesized from a thermal decomposition process have been reported by Schaak and co‐workers for HER.^[^ ^49^ ^]^ One‐pot pyrolysis reaction is applied to transform nickel acetylacetonate into Ni_2_P without additional templates. Transmission electron microscope (TEM) image clearly demonstrates the well‐defined internal void within these Ni_2_P quasi‐spherical nanoparticles (**Figure** **2**a). A close inspection confirms these single crystalline particles exhibit a high density of exposed (001) facets (Figure 2b). The hollow structured Ni_2_P film delivers high HER activity with small overpotentials (*η*) of 130 and 180 mV to reach the cathodic current densities (*j*) of 20 and 100 mA cm^–2^ (Figure 2c). Tafel plots indicate the Ni_2_P sample has an exchange current density of 3.3 × 10^–5^ A cm^–2^ with a Tafel slope of ≈46 mV dec^−1^ (Figure 2d). Although the HER activity of Ni_2_P hollow sphere is still inferior to Pt with negligible *η*, its enhanced performance indicates the potential of hollow structures for advanced HER catalysts.

#### 2.1.2 Hierarchical Hollow Electrocatalysts

Compared with the simplest hollow spheres, hierarchical hollow structures composed of interconnected 1D or 2D building blocks are favorable for water splitting, because these high‐energy surfaces could bring more exposed active sites. For example, Lou and co‐workers have reported the construction of hierarchical *β*‐Mo_2_C nanotubes from 2D nanosheets via the carbonization of a Mo‐polydopamine precursor.^[^ ^50^ ^]^ Field‐emission scanning electron microscopy (FESEM) image shows that the tubular‐like *β*‐Mo_2_C structures are built from ultrathin nanosheets (Figure 2e). TEM investigation confirms the hollow nature of these nanotubes (Figure 2f). Compared with the *β*‐Mo_2_C nanoflowers, these hierarchical nanotubes exhibit superior HER activity with smaller *η* (197 mV for nanotube vs 220 mV for nanoflowers) to reach the *j* of 20 mA cm^–2^ in acid (Figure 2g). Furthermore, the presence of certain promoter cations could decrease the binding energy of hydrogen for improved intrinsic property. Yu et al. reported the formation of a series of single‐shelled M‐MoS_3_ (M = Co, Ni) hollow structures with tailorable morphologies using water‐soluble precursors.^[^ ^51^ ^]^ Through a fast self‐templated precipitation reaction, a single‐shelled hollow structure of M‐MoS_4_ sample with a smooth surface is obtained (Figure 2h). A subsequent annealing treatment endows the resultant M‐MoS_3_ hollow electrocatalysts with better HER performance compared with the bulk MoS_2_ particles. In another case, hierarchical MoS_2_ nanoboxes with incorporated nickel and cobalt (Ni‐Co‐MoS_2_) have been obtained via the solvothermal reaction between nickel‐cobalt Prussian‐blue analog (Ni‐Co‐PBA) and (NH_4_)_2_MoS_4_.^[^ ^52^ ^]^ This template‐engaged reaction changes the solid Ni‐Co‐PBA nanocubes into hierarchical nanoboxes assembled by ultrathin Ni‐Co‐MoS_2_ nanosheets (Figure 2i). The hierarchical nanoboxes reveal structural and compositional superiorities over bare MoS_2_ nanosheets with more electroactive sites and faster Faradaic kinetics. As expected, Ni‐Co‐MoS_2_ nanoboxes require only a *η* of 155 mV to deliver a current density of 10 mA cm^–2^, much smaller than that required for the bare MoS_2_ (Figure 2j).

**Figure 3:**
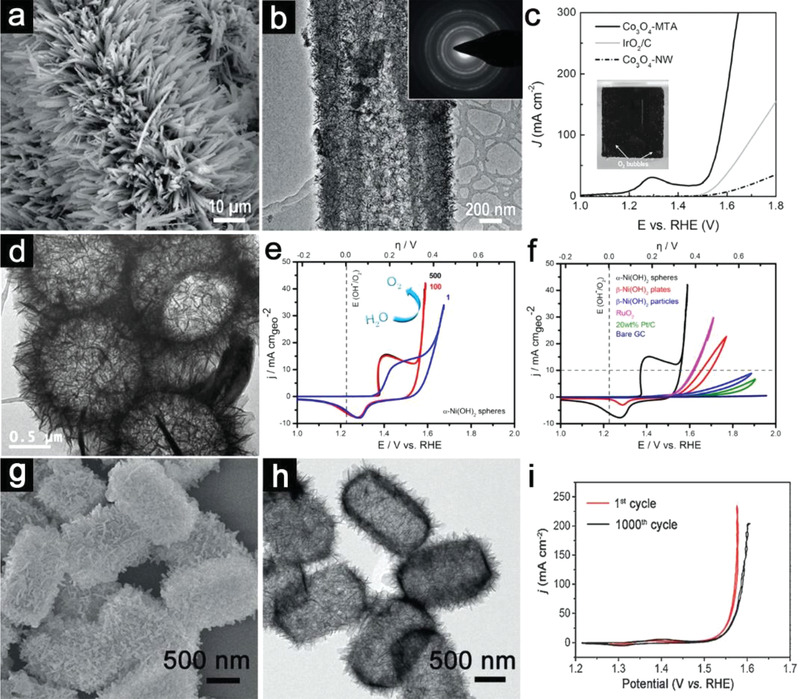
a) FESEM and b) TEM images of Co_3_O_4_‐MTA. c) LSV curves of Co_3_O_4_‐MTA and control samples. The inset in (c) is a digital photo of Co_3_O_4_‐MTA as the direct working electrode for OER. (a–c) Reproduced with permission.^[^ ^54^ ^]^ Copyright 2017, Wiley‐VCH. d) TEM image and e) CV curves recorded at different cycles for *α*‐Ni(OH)_2_ hollow spheres. f) Comparison of CV curves recorded at 100th cycle for *α*‐Ni(OH)_2_ hollow spheres and other catalysts. (d–f) Reproduced with permission.^[^ ^55^ ^]^ Copyright 2014, American Chemical Society. g) FESEM, h) TEM images, and i) CV curves of Ni‐Fe LDH hollow prisms. (g–i) Reproduced with permission.^[^ ^56^ ^]^ Copyright 2018, Wiley‐VCH.

Single‐shelled hollow structures also show great potential as direct OER catalysts. Different from the noble metal used in HER, metal oxides are preferred in OER to endure the oxidation process. As an ideal OER catalyst, IrO_2_ has theoretically reasonable binding energies with reaction intermediates. However, the high cost and poor cycling stability stimulate the research for appropriate alternatives. Specifically, transition metal (Mn, Co, Ni, Fe) based oxides with variable valence state have been widely investigated as candidates for OER.^[^ ^8^, ^53^ ^]^ For instance, Qiao and co‐workers have reported the design of hollow Co_3_O_4_ microtube array (MTA) with hierarchical macro‐/mesoporosity as OER catalysts.^[^ ^54^ ^]^ Cobalt hydrogen phosphate (CoHPO_4_) microwires grown on Ni foam are selected as the starting templates. The obtained Co_3_O_4_ sample from the potentiostat treatment inherits 1D morphology of the CoHPO_4_ template (**Figure** **3**a). As revealed by time‐dependent microscopy studies, the smooth surface of CoHPO_4_ is gradually changed into a hierarchical shell with the simultaneous consumption of the solid core (Figure 3b). Linear sweep voltammetry (LSV) polarization curves illustrated the OER activity of Co_3_O_4_ MTA could significantly surpass that of the solid Co_3_O_4_ nanowire array (Figure 3c). More importantly, the Co_3_O_4_ microtube array could induce higher current than IrO_2_/C in the whole operating potential window, further revealing the importance of structural control. Besides the metal oxides, a series of metal hydroxides/(oxy)hydroxides/phosphides are also considered as possible OER electrocatalysts. Yan's group has done a pioneering work about the preparation of *α*‐Ni(OH)_2_ hollow spheres for efficient OER catalysis from the template‐free strategy.^[^ ^55^ ^]^ As shown in Figure 3d, the obtained hollow *α*‐Ni(OH)_2_ spheres are composed of interconnected nanosheets. Cyclic voltammetry (CV) curves indicate the activation of *α*‐Ni(OH)_2_ into electroactive species *γ*‐NiOOH from the 1^st^ cycle to 100^th^ cycle (Figure 3e). The activity comparison between different samples demonstrates the superiority of *α*‐Ni(OH)_2_ hollow spheres over *β*‐Ni(OH)_2_ solid nanoplates and nanoparticles (Figure 3f). Compared with the hollow structures of monometallic compounds, mixed metal compounds might provide another dimensionality to optimize the water oxidation performance. For example, Yu et al. reported the formation of hierarchical Ni‐Fe layered double hydroxide (LDH) hollow nanoprisms with tailorable Ni/Fe atomic ratio.^[^ ^56^ ^]^ A facile self‐templated approach has been utilized to change the solid nickel precursors into hollow prisms composed of ultrathin Ni‐Fe LDH nanosheets (Figure 3g,h). As shown in Figure 3i, Ni‐Fe LDH hollow prisms demonstrate excellent OER activity with high stability over 1000 cycles. The origin of enhanced performance might be due to the desirable compositions and more accessible active sites.

### 2.2 Multi‐Shelled Hollow Structures as Direct Electrocatalysts

The construction of multi‐shelled hollow structures for direct electrocatalysts is a powerful strategy to improve electrocatalytic performance, however, synthetic difficulties and concerns about mass/electron transport add uncertainty of this approach. Compared with the single‐shelled hollow structures, hollow electrocatalysts with multi‐shelled feature exhibit apparent superiorities for electrocatalysis. Multi‐shelled hollow structures have higher surface area and better utilization of the inner space. Moreover, the interlayers can support each other for enhanced mechanical stability.^[^ ^25^, ^30^ ^]^ Therefore, it can be expected the multi‐layered hollow electrocatalysts could have improved catalytic activity per unit area with better cycling stability. Nevertheless, the design of multi‐layered hollow electrocatalysts always requires trade‐offs between shell number and mass/electron transport. Besides, complex synthetic route is another concern for the practical application of multi‐shelled hollow electrocatalysts for water splitting.

**Figure 4:**
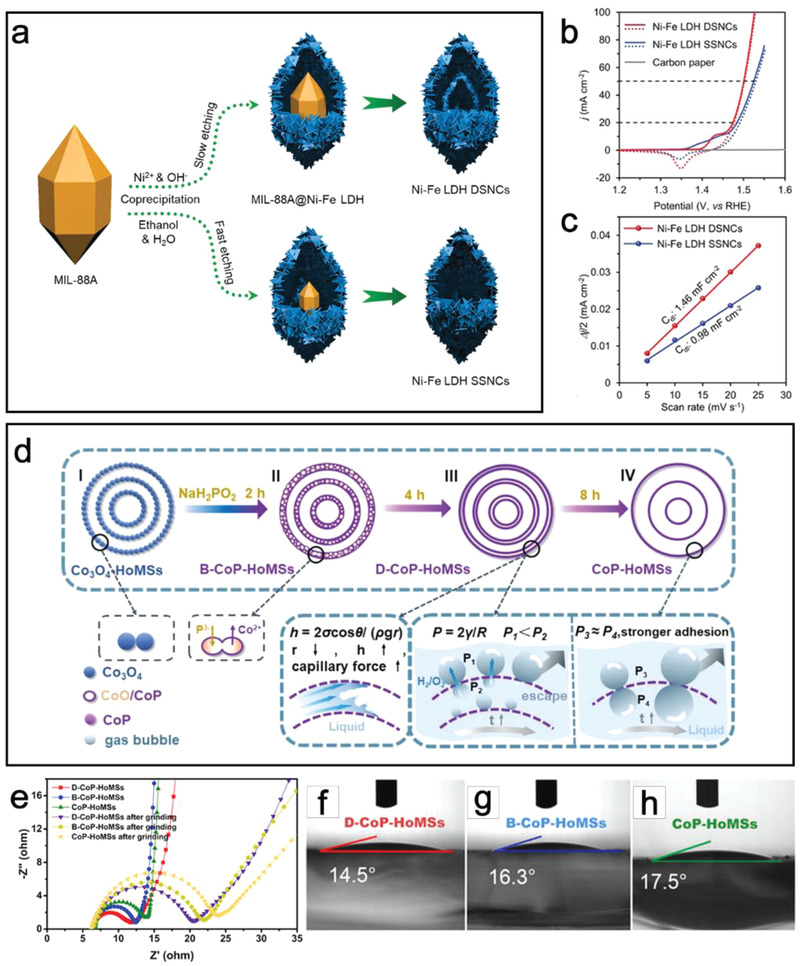
a) Schematic illustration of the formation of Ni‐Fe LDH DSNCs and SSNCs. b) CV curves of Ni‐Fe LDH electrocatalysts. c) Half of the capacitive current density (Δ*J*/2) as a function of the scan rate for Ni‐Fe LDH DSNCs and SSNCs. (a–c) Reproduced with permission.^[^ ^60^ ^]^ Copyright 2020, Wiley‐VCH. d) Illustration of the formation process of CoP HoMSs with different micro‐nanostructure of shells. e) Nyquist plots of HoMSs samples. (f–h) Water contact angle images of HoMSs. (d–h) Reproduced with permission.^[^ ^27^ ^]^ Copyright 2021, Wiley‐VCH.

Recently, the development of self‐engaged template approaches brings inspiring advances toward this powerful strategy.^[^ ^57^, ^58^ ^]^ Self‐templated methods have enriched the formation of multi‐shelled hollow structures based on heterogenous contraction, etching, and diffusion mechanisms. Benefitting from tunable structure and chemical compositions, metal‐organic frameworks (MOFs) assembled by metal ions/clusters with organic ligands have been regarded as ideal templates for multi‐shelled hollow structures.^[^ ^28^, ^59^ ^]^ Lou and co‐workers reported a simultaneous etching and coprecipitation method to obtain Ni‐Fe LDH nanocages with different shells (**Figure** **4**a).^[^ ^60^ ^]^ In this work, MIL‐88A (Materials from Institut Lavoisier) particles are selected as the sacrificial templates. By regulating the ratio of ethanol to water in the mixed solution, the Ni‐Fe LDH shell number can be delicately controlled. Concretely, more water could lead to the fast etching of MIL‐88A, thus forming single‐shelled nanocages (SSNCs), while more ethanol is responsible for constructing double‐shelled nanocages (DSNCs) due to the slow etching rate. Energy‐dispersive X‐ray (EDX) spectrum of Ni‐Fe LDH DSNCs reveals Ni and O elements concentrate in the inner layer while Fe element is in the outer shell, suggesting different distributions of active components throughout the whole DSNCs. Electrochemical measurements demonstrate that Ni‐Fe LDH DSNCs with effective surface area exposure exhibit superior OER activity and double‐layer capacitance (*C*_dl_) to that of single‐shelled one (Figure 4b,c).

Apart from the modification of active components, multi‐shelled hollow structures could also guarantee tunable mass transport. As depicted in Figure 4d, Wang and co‐workers have delicately fabricated CoP hollow multi‐shell structures (HoMSs) with different micro‐nanostructure of shells.^[^ ^27^ ^]^ The formation of bubble‐like structure can be ascribed to the difference between rapid outward diffusion of Co species in Co_3_O_4_ and slowly inward diffusing of P species, causing a cavity inside the nanoparticles. By controlling phosphorization time, the structure evolves from bubble‐like (B‐CoP‐HoMSs) to close duplicated shells with a narrow spacing (D‐CoP‐HoMSs) and finally turns into a solid one (CoP‐HoMSs). Electrochemical impedance spectroscopy shows that D‐CoP‐HoMSs possess the smallest semicircle in Nyquist plots, suggesting the faster charge‐transport than B‐CoP‐HoMSs and CoP‐HoMSs (Figure 4e). Besides, D‐CoP‐HoMSs also demonstrate greater hydrophilicity (Figure 4f–h) and larger gas contact angle, which is favorable for rapid gas release and liquid diffusion because of unbalanced Laplace pressure, thus achieving quick HER and OER kinetics.

### 2.3 Frame‐Like Hollow Structures as Direct Electrocatalysts

The design of highly open hollow materials has been proven as an efficient approach for direct electrocatalysts to expose a high percentage of active surfaces per a given mass. Hollow materials with frame‐like or skeleton‐like features have been considered as intriguing designs for electrocatalysts. These open architectures could retain all reactive corners and edges and alter the density of low‐coordination atoms on surface.^[^ ^24^, ^45^, ^61^ ^]^ In addition, the majority atoms within open hollow catalysts could participate reactions except for those located at the corner sites.

#### 2.3.1 Alloy‐Based Nanoframes

**Figure 5:**
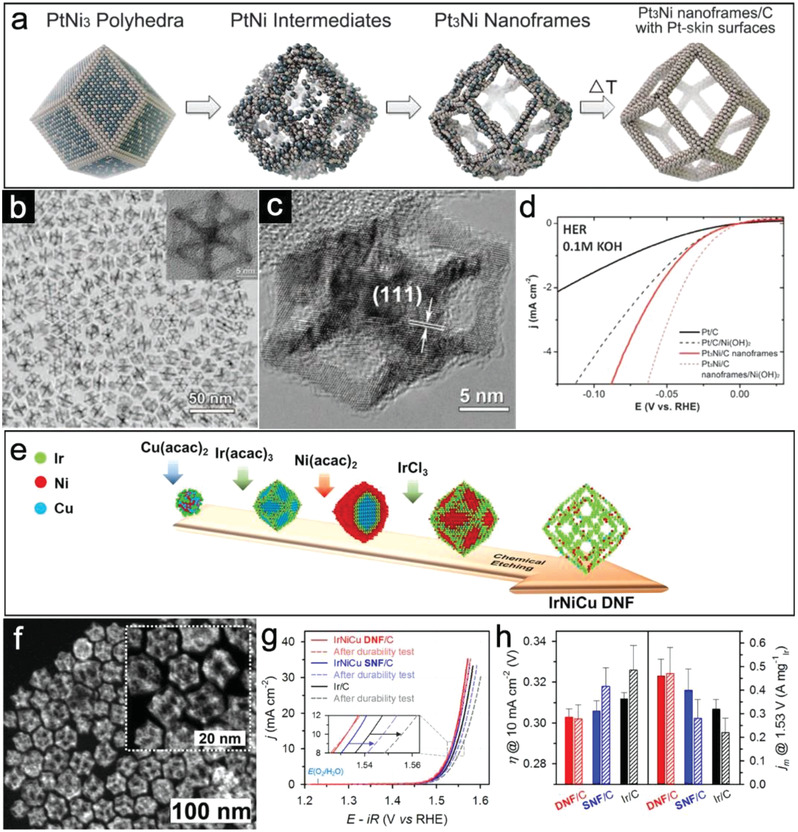
a) Schematic illustration of the formation of hollow Pt_3_Ni nanoframes. b) TEM and c) HRTEM images of Pt_3_Ni nanoframes. d) HER performances of Pt/C, Pt/Ni(OH)_2_/C, Pt_3_Ni nanoframes/C, and Pt_3_Ni frames/Ni(OH)_2_/C in alkaline electrolyte. (a–d) Reproduced with permission.^[^ ^40^ ^]^ Copyright 2014, The American Association for the Advancement of Science. e) Schematic illustration of the formation of IrNiCu DNF structures. f) HAADF‐STEM image of DNF. Inset: Enlarged HAADF‐STEM image. g) OER activities for IrNiCu DNF/C, IrNiCu SNF/C, and commercial Ir/C (20 wt% Ir) catalysts. h) *η* to drive 10 mA cm^−2^ and Ir mass activity (*j*_m_) at 1.53 V (vs RHE) of the catalysts before (bar) and after durability test (pattern bar). (e–h) Reproduced with permission.^[^ ^62^ ^]^ Copyright 2017, American Chemical Society.

In this context, the design and construction of noble metal‐based alloys with open hollow nanostructures could efficaciously control dosage and reduce cost. More importantly, open feature could enable fast access of reactant molecules. As a typical example, Chen et al. have reported the formation of Pt_3_Ni nanoframes via an in situ interior erosion reaction.^[^ ^40^ ^]^ This unique self‐engaged etching method involves the simultaneous phase transition and structure evolution processes from PtNi_3_ solid polyhedrons to Pt_3_Ni nanoframes (**Figure** **5**a). TEM and high‐resolution TEM (HRTEM) characterizations show the high uniformity of these single crystalline hollow frames (Figure 5b,c). Owing to the open structure and desirable surface composition, the HER performance of Pt_3_Ni nanoframes in KOH is much better than that of commercial Pt/C (Figure 5d). After electrochemically depositing Ni(OH)_2_ clusters on surface, their HER activity is further enhanced due to the easy dissociation of water. In another interesting example, Park et al. reported a more complex open hollow structure with a double‐layered nanoframe (DNF) feature.^[^ ^62^ ^]^ By delicately balancing the formation kinetics of Ir and transition metal (Ni and Cu) precursors, a core–shell‐type alloy@alloy structure could be achieved in the one‐pot synthesis (Figure 5e). After the selective etching process, the high‐angle annular dark‐field scanning TEM (HAADF‐STEM) image reveals that IrNiCu DNF nanostructure with a rhombic dodecahedral morphology is formed (Figure 5f). This unique open structure provides the IrNiCu DNF/C sample with much more electrocatalytic active sites compared with single‐shelled IrNiCu nanoframe and Ir/C catalysts, leading to the lowest *η* to drive OER in acidic condition with high durability (Figure 5g,h).

As an emerging type of advanced functional materials, high‐entropy alloys (HEAs) are composed of four or more metal elements.^[^ ^63^, ^64^ ^]^ In view of the unique properties like high entropy effect, large lattice distortion, and cocktail effect, high‐entropy materials (HEMs) derived from HEAs manifest great potential as promising electrocatalysts, such as FeNiCoCrMnS_2_^[^ ^65^ ^]^ and CoFeNiMnMoPi.^[^ ^66^ ^]^ It is believed that the combination of hollow structure with HEMs can shed some light on the design of efficient water‐splitting electrocatalysts.

#### 2.3.2 MOF‐Derived Nanoframes

**Figure 6:**
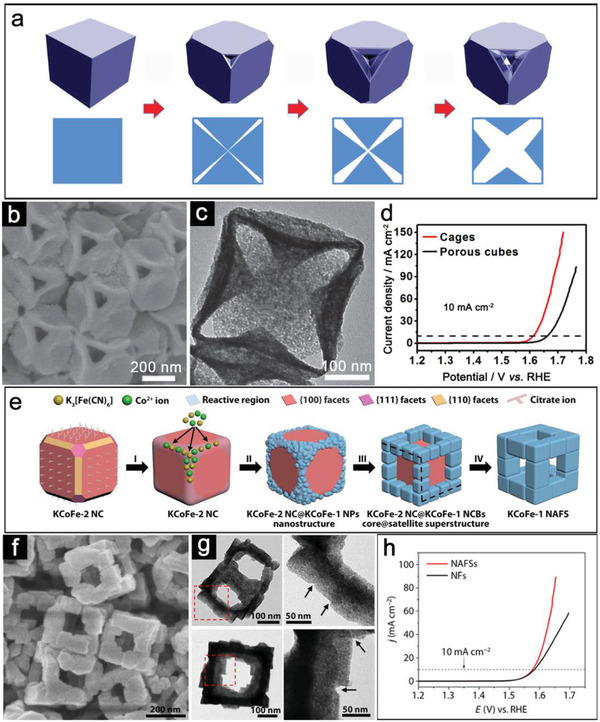
a) Schematic illustration of the formation of the Ni‐Co PBA cages. b) FESEM and c) TEM images of mixed oxide cages. d) OER performance of the Ni‐Co PBA cages and porous cubes. (a–d) Reproduced with permission.^[^ ^68^ ^]^ Copyright 2016, Wiley‐VCH. e) Schematic illustration of the formation of KCoFe‐1 NAFSs. f) FESEM and g) TEM images of Co‐Fe mixed oxides derived from KCoFe‐1 NAFSs. h) LSV curves of Co‐Fe‐O NAFSs and NFs. (e–h) Reproduced with permission.^[^ ^67^ ^]^ Copyright 2017, The American Association for the Advancement of Science.

The construction of frame‐like structure has also demonstrated the superiority in water splitting for nonalloy catalysts, although further understandings of formation mechanism are still needed.^[^ ^67^ ^]^ For example, cage‐like open nanostructures of Ni‐Co mixed oxide are derived from Ni‐Co Prussian‐blue‐analog (PBA) nanocages consisting of pyramidal walls.^[^ ^68^ ^]^ The structural evolution of the Ni‐Co PBA precursors from nanocubes to nanocages can be described as a controlled etching process along the diagonal with the assistance of internal defects (**Figure** **6**a). After the annealing treatment in air, the obtained porous Ni‐Co mixed oxide inherits open features from the Ni‐Co PBA nanocage (Figure 6b,c). By virtue of open architecture, Ni‐Co mixed oxide cages require a smaller potential of 1.61 V versus reversible hydrogen electrode (vs RHE) to drive 10 mA cm^−2^ compared with the porous cubes (1.66 V vs RHE) for OER (Figure 6d). Starting from Co‐Fe PBA (atomic ratio of K/Co/Fe = 0.67:1:0.71, denoted as KCoFe‐1) nanocuboids assembled frame‐like superstructure (NAFSs), Nai et al. realized the formation of Co‐Fe mixed oxides with open frame‐like superstructures.^[^ ^67^ ^]^ An oriented assembly (OA) strategy is developed for the structural evolution. Figure 6e illustrates the formation process of KCoFe‐1 NAFSs, which involves the phase transition from KCoFe‐2 (atomic ratio of K/Co/Fe = 0.07:1:0.67) to KCoFe‐1, epitaxial growth, confined assembly, and OA steps. After the oxidation treatment, the derived Co‐Fe oxide sample is highly porous with similar frame‐like hollow structures from the KCoFe‐1 NAFS precursor (Figure 6f,g). When evaluated as catalysts, the Co‐Fe oxide transformed from KCoFe‐1 NAFSs shows enhanced OER activity compared with the derived sample from KCoFe‐3 (atomic ratio of K/Co/Fe = 0.97:1:0.84) nanoframes (NFs). Although these two samples require similar overpotentials (340 mV vs 350 mV) to afford the current density of 10 mA cm^–2^, LSV curves indicate the Co‐Fe oxide NAFS sample has faster reaction kinetic compared with Co‐Fe oxide NFs (Figure 6h). Furthermore, highly active nanoframes derived from MOFs could enable the integration of bifunctional electrocatalysts into one electrode design. As a typical example, Mu and co‐workers developed a unit‐exchange strategy between [MoO_4_] in (NH_4_)_6_Mo_7_O_24_ and [Co‐N*_x_* C*_y_*] units in Co PBA.^[^ ^69^ ^]^ After subsequent pyrolysis, the resultant Co_3_O_4_‐Mo_2_N nanoframes inherit the functionalities of individual components and demonstrate outstanding activities toward both HER and OER.

## 3 Heterostructured Electrocatalysts

Recently, heterostructured materials have exhibited great potential in electrochemical water splitting, which are generally composed of active electrocatalyst and nonactive substrate.^[^ ^70^, ^71^ ^]^ Other than being as the direct electrocatalysts, highly conductive hollow structures could also serve as hosts in heterostructured electrocatalysts to prevent aggregation of active species or introduce new functions, due to their high surface area and low tap density. The uniform distribution and strong binding with conductive substrates enable the electroactive species better stability and reduced resistive loss. These heterostructured electrocatalysts could be divided into two categories based on distinct or vague spatial position of the active species in hollow structures. It should also be noted that the spatial allocation of catalytic components is vital for the interactions between components.^[^ ^21^ ^]^ In particular, for the heterostructured electrocatalysts with distinct spatial position, the active components are confined into the interior void space, porous shells, or supported on the surfaces of hollow hosts. Interior cavities significantly decrease the loading of active components per unit area, while porous shells guarantee the tunable mass transport of heterostructured electrocatalysts.^[^ ^27^ ^]^ In other cases, the hollow materials also act as secondary electrocatalysts or promoters to facilitate tandem reactions together with their entrapped active components.^[^ ^25^ ^]^

**Figure 7:**
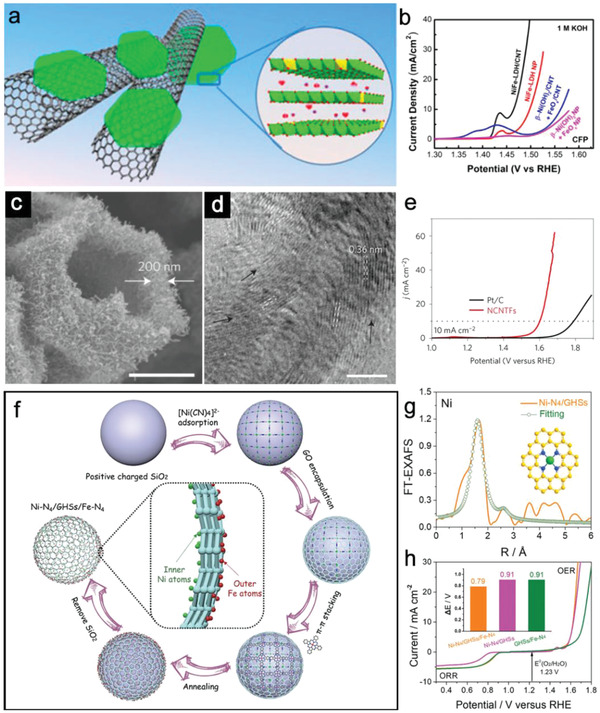
a) Schematic illustration of the Ni‐Fe‐LDH/CNT hybrid architecture. b) OER activities of Ni‐Fe‐LDH/CNT hybrid loaded on CFP and control groups in KOH. (a,b) Reproduced with permission.^[^ ^72^ ^]^ Copyright 2013, American Chemical Society. c) FESEM and d) HRTEM images of NCNTFs. e) OER activities of NCNTFs and Pt/C. Scale bars: c) 1 µm; d) 5 nm. (c–e) Reproduced with permission.^[^ ^73^ ^]^ Copyright 2016, Nature Publishing Group. f) Synthetic process of the Ni‐N_4_/GHSs/Fe‐N_4_ structure. g) EXAFS fitting curves of Ni‐N_4_/GHSs. h) Overall polarization curves of Ni‐N_4_/GHSs/Fe‐N_4_. (f–h) Reproduced with permission.^[^ ^74^ ^]^ Copyright 2020, Wiley‐VCH.

For the heterostructured electrocatalysts with well‐defined spatial position, conductive carbonaceous materials with hollow features such as carbon nanotubes (CNTs) have been widely used as hosts for various heterostructured electrocatalysts. As a typical example, multi‐walled CNTs (MWCNTs) have been utilized as substrates for the growth of Ni‐Fe LDH nanoplates through a solvothermal treatment.^[^ ^72^ ^]^ As illustrated in **Figure** **7**a, ultrathin Ni‐Fe LDH nanoplates decorated with FeO*_x_* nanoparticles are introduced as electrocatalytically active species onto the surface of MWCNTs. X‐ray absorption near edge structure measurements show strong interaction effects of Ni‐Fe‐LDH nanoplates and MWCNTs, indicating the facilitated charge transfer. As a result, the Ni‐Fe‐LDH/CNT hybrid catalyst exhibits smaller *η* and faster reaction kinetics for OER compared with control samples (Figure 7b). Interestingly, carbon materials could also serve as the direct electrocatalyst and the loaded materials such as metal or metallic compounds could play as structural agent for the growth of CNT. For example, Xia et al. reported the hollow N‐doped carbon nanotube frameworks (NCNTFs) using zeolitic imidazolate framework (ZIF‐67) as the self‐template by direct pyrolysis in a reductive atmosphere (Figure 7c).^[^ ^73^ ^]^ Co nanoparticles are completely encapsulated within the tip of the multi‐walled CNTs, and inaccessible to reactants. HRTEM image reveals that the graphitic layers are not perfectly parallel to the axis direction of the CNTs, generating more exposed edges (Figure 7d). Due to the characteristics of composition and structure, the optimized NCNTFs obtained at 700 ℃ could efficiently catalyze the OER with higher electrocatalytic activity than that of commercial Pt/C in alkaline solution (Figure 7e). Apart from the above types of combination, hollow nanostructures could also serve as the host materials to load single‐atom catalysts (SACs). The porous structures with good stability and confined microenvironment would be ideal supports to stabilize SACs. Figure 7f shows a typical example of Ni and Fe SACs on the inner and outer walls of graphene hollow nanospheres (denoted as Ni‐N_4_/GHSs/Fe‐N_4_) using a step‐by‐step self‐assembly strategy.^[^ ^74^ ^]^ The extended X‐ray absorption fine structure (EXAFS) fitting analysis suggests the presence of atomically dispersed Ni‐N_4_ moieties in Ni‐N_4_/GHSs and Fe‐N_4_ moieties in GHSs/Fe‐N_4_, which are responsible for OER and oxygen reduction reaction, respectively (Figure 7g). These Janus hollow structures with high conductivity could largely utilize the electrocatalytically active sites and enable fast electron transport. As a result, the as‐prepared Ni‐N_4_/GHSs/Fe‐N_4_ electrocatalyst exhibits a low potential gap (Δ*E*) value of about 0.79 V, superior to that of Ni‐N_4_/GHSs and GHSs/Fe‐N_4_ (Figure 7h).

**Figure 8:**
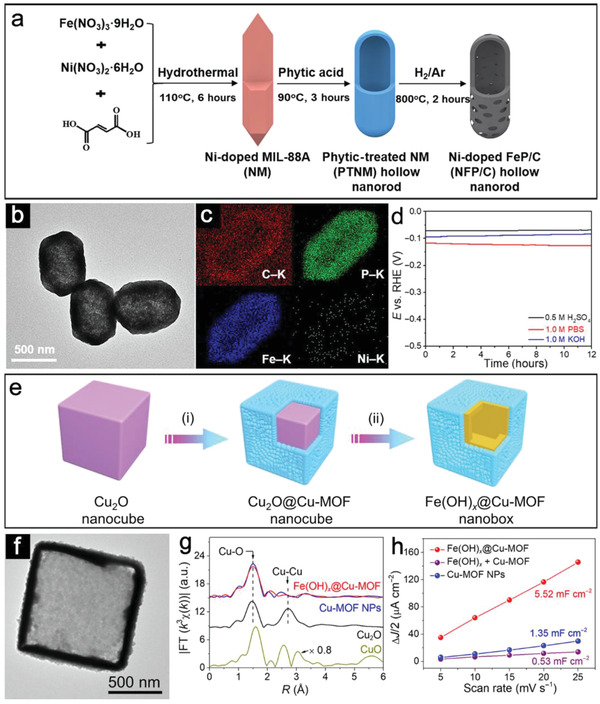
a) Schematic illustration of the formation of NFP/C hollow nanorods. b) TEM and c) EDX mapping images of NFP/C hollow nanorods. d) Durability test of NFP/C‐3 in acidic, neutral, and alkaline solutions. Reproduced with permission.^[^ ^76^ ^]^ Copyright 2019, The American Association for the Advancement of Science. e) Schematic illustration of the synthetic process for Fe(OH)*_x_* @Cu‐MOF nanobox. f) TEM image of Fe(OH)*_x_* @Cu‐MOF. g) The Fourier transform curves of Cu K‐edge EXAFS spectrum for Fe(OH)*_x_* @Cu‐MOF NBs. h) Δ*J*/2 as a function of the scan rate for Fe(OH)*_x_* @Cu‐MOF. Reproduced with permission.^[^ ^77^ ^]^ Copyright 2021, The American Association for the Advancement of Science.

Another category of the heterostructured electrocatalysts is the integrated hollow nanoreactors, where spatial positions for electrocatalyst and support in hollow structures are rather vague. In this design, hollow hosts could protect active species from aggregation or direct corrosion during water‐splitting reactions. Besides, it is well known that the practical electrocatalytic performance is highly sensitive to the surface/interface properties of electrocatalysts. Therefore, to achieve significantly promoted activities, some surface modification strategies have been developed, including heterojunction design, defect control, element doping, and interface engineering.^[^ ^75^ ^]^ For instance, Lu et al. reported an effective method to generate Ni‐doped FeP/carbon (NFP/C) hollow nanorods with tailorable length and composition based on self‐engaged etching and coordination reactions.^[^ ^76^ ^]^ Both the carbon and Ni‐doped FeP species are derived from the thermal treatment of the phytic acid‐treated MIL‐88A (**Figure** **8**a). TEM image reveals the rod‐like morphology of these single‐shelled hollow structures (Figure 8b). EDX mapping results show the carbon element is well dispersed within the whole particle (Figure 8c). Carbon could support the Ni‐doped FeP to avoid collapse during calcination. More importantly, the carbon species play a great role for maintaining the structural integrity and catalytic performance in acidic, neutral, and alkaline solutions (Figure 8d). Furthermore, the integrated hollow nanoreactors could also introduce functional species. In a recent study, Lou and co‐workers have delicately fabricated a hybrid electrocatalyst of conductive Cu‐MOF supported on Fe(OH)*_x_* nanobox.^[^ ^77^ ^]^ This unique design is realized via a self‐templated solvothermal reaction followed by redox‐etching strategy, as described in Figure 8e. TEM image of an individual Fe(OH)*_x_* @Cu‐MOF hollow nanobox elucidates that the ultrathin Cu‐MOF layer is tightly coated on the surface of the Fe(OH)*_x_* shell, which cannot be clearly distinguished (Figure 8f). EXAFS reveals the presence of coordinatively unsaturated Cu_1_‐O_2_ centers due to the ultrathin feature of the Cu‐MOF layer (Figure 8g). As a result, Fe(OH)*_x_* @Cu‐MOF manifests a much larger *C*_dl_ value of 5.52 mF cm^–2^, which is approximately four times that of Cu‐MOF (Figure 8h). The incorporated unsaturated Cu_1_‐O_2_ sites thermodynamically favor the *H formation toward fast HER kinetics.

## 4 Beyond Water Splitting

**Figure 9:**
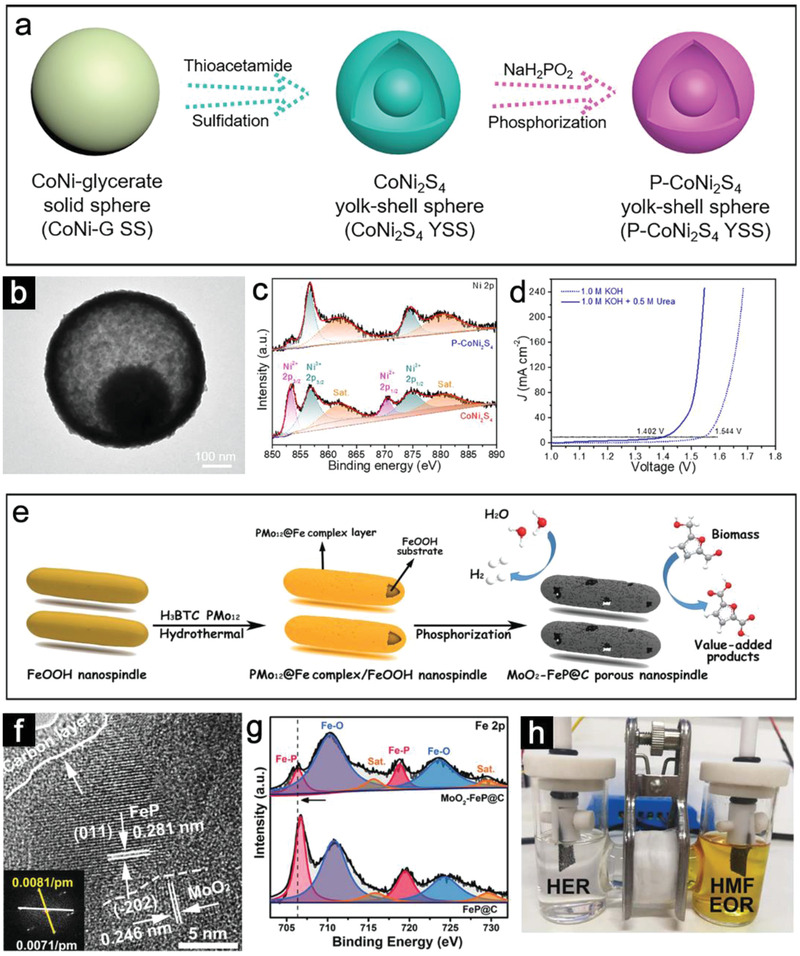
a) Schematic illustration of the formation of P‐CoNi_2_S_4_ YSSs. b) TEM image of P‐CoNi_2_S_4_ YSSs. c) High‐resolution XPS spectra of Ni 2p of P‐CoNi_2_S_4_ YSSs. d) LSV plots of P‐CoNi_2_S_4_ YSSs toward water splitting and urea electrolysis. Reproduced with permission.^[^ ^80^ ^]^ Copyright 2021, John Wiley and Sons. e) Schematic illustration of the synthesis of the MoO_2_‐FeP@C porous nanospindle for HER and HMFEOR. f) HRTEM image of MoO_2_‐FeP@C. g) High‐resolution XPS spectra of Ni 2p of MoO_2_‐FeP@C. h) Demonstration of the MoO_2_‐FeP@C couple used in an H‐type cell. Reproduced with permission.^[^ ^84^ ^]^ Copyright 2020, John Wiley and Sons.

Beyond normal OER and HER, a recent trend for the design of electrocatalysts in water splitting is to replace OER by a more thermodynamically favorable oxidation reaction to reduce the energy consumption.^[^ ^7^ ^]^ In this innovative strategy, the explosion risk from H_2_/O_2_ mixing could be avoided and a much lower cell voltage is required for the hydrogen generation with reduced energy input. Urea oxidation reaction with a low theoretical voltage of 0.37 V (vs RHE) has been reported as one of the promising candidates to replace OER.^[^ ^78^, ^79^ ^]^ In a typical example, **Figure** **9**a shows the construction of the porous phosphorus‐substituted CoNi_2_S_4_ yolk–shell spheres (P‐CoNi_2_S_4_ YSSs) for both overall water splitting (OWS) and urea‐assisted water splitting to produce hydrogen.^[^ ^80^ ^]^Figure 9b reveals the porous P‐CoNi_2_S_4_ YSSs with a thin shell composed of ultrafine nanoparticles, which is beneficial for exposing more active sites. X‐ray photoelectron spectroscopy (XPS) results suggest that more electronic interactions have occurred on the P‐CoNi_2_S_4_, generating enriched Ni^3+^ content, which promotes stronger redox reaction and enhances electrocatalytic performance (Figure 9c). When assembled using the P‐CoNi_2_S_4_ at both anode and cathode, the urea‐mediated electrolyzer exhibits a rather low potential of 1.402 V to achieve 10 mA cm^–2^, which is 142 mV less than that for water splitting (Figure 9d).

Furthermore, replacing OER with a more thermodynamically favorable organic oxidation reaction could produce value‐added fine chemicals at the anode simultaneously, instead of O_2_, N_2_, CO_2_ or other less valuable species.^[^ ^81^ ^]^ The type of organic substrates is critical for electrosynthesis of chemicals with high yield and selectivity when coupled with HER. At present, alcohols, aldehydes, amines, and carbohydrates are considered as ideal anodic species to substitute OER.^[^ ^81^, ^82^, ^83^ ^]^ For example, as shown in Figure 9e, Fu and co‐workers have reported a porous carbon‐encapsulated MoO_2_‐Fe (MoO_2_‐FeP@C) heterojunction nanospindle using FeOOH as a self‐sacrificial template for both HER and 5‐hydroxymethylfurfural (HMF) electrooxidation reaction (EOR).^[^ ^84^ ^]^As verified by HRTEM, the porous structure enables the direct contact between the inner MoO_2_‐FeP heterojunction with reactants (Figure 9f). Moreover, the XPS results verify the electron redistribution between MoO_2_ and FeP occurring at the interface (Figure 9g). Benefitting from these features, the electrolysis cell using MoO_2_‐FeP@C performs well for both cathodic hydrogen and anodic 2,5‐furandicarboxylic acid (FDCA) production (Figure 9h). Interestingly, 3D vanadium nitride (VN) and Pd/VN hollow nanospheres were delicately designed by Wang and co‐workers to generate value‐added products at both electrodes, where HMF was converted to FDCA by VN at the anode and to 2,5‐bishydroxymethyl‐tetrahydrofuran (DHMTHF) by Pd/VN at the cathode.^[^ ^85^ ^]^Again, this work demonstrates the innovative application of hollow electrocatalysts beyond water splitting.

Considering the cost‐effective storage and transport of H_2_, many researchers have proposed the water splitting coupled with CO_2_ hydrogenation reaction, which directly converts the H_2_ during water electrolysis into value‐added chemicals. Typically, hollow or core–shell catalysts provide promising solutions to improve the Faradaic efficiency (FE) for the desired products.^[^ ^86^ ^]^ For example, a catalyst composed of core–shell Cu_2_O/Cu nanoparticles anchored in N‐doped porous carbon (Cu_2_O/Cu@NC) was reported for electrochemical CO_2_‐to‐formate conversion, achieving a maximum FE of 70.5% at −0.68 V versus RHE.^[^ ^87^ ^]^ The high FE can be ascribed to the advanced hollow structure, high N content, and well‐dispersed Cu_2_O/Cu species.

## 5 Conclusion and Perspectives

**Figure 10:**
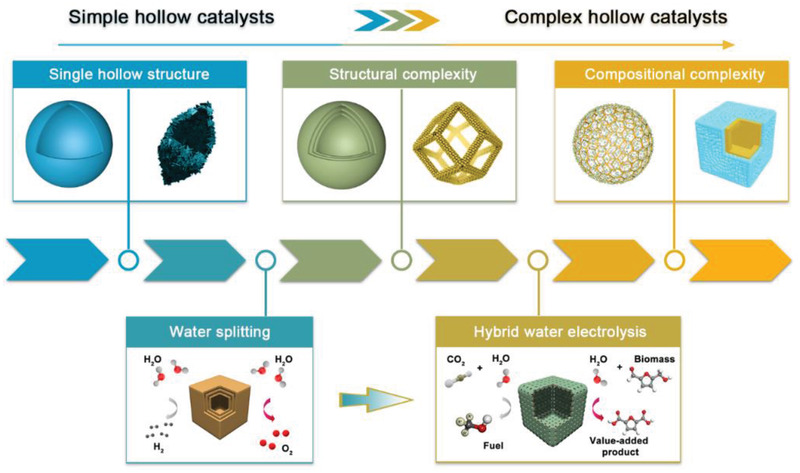
The advances of hollow electrocatalysts toward water splitting and beyond. Reproduced with permission.^[^ ^60^ ^]^ Copyright 2020, John Wiley and Sons. Reproduced with permission.^[^ ^40^ ^]^ Copyright 2014, The American Association for the Advancement of Science. Reproduced with permission.^[^ ^77^ ^]^ Copyright 2021, The American Association for the Advancement of Science.

Electrochemical water splitting serves as a promising pathway to produce hydrogen as a future fuel. The employment of hollow structured electrocatalysts has aroused tremendous interest as an advanced approach for enhanced hydrogen and oxygen production performances, owing to their advantageous features, such as large surface area and reduced mass/charge transfer path. Besides, the tunable shell architecture and internal construction bring great opportunities in the structural design and composition modification to achieve optimized electrocatalytic activities. Considering different requirements for HER/OER and diverse roles of hollow structures, design principles of advanced hollow electrocatalysts are quite distinctive. In this perspective, we summarize some recent developments in the design of hollow nanostructures as direct electrocatalysts and supporting hosts for electrochemical water splitting and beyond. The schematic summary of the advances of the hollow electrocatalysts toward water splitting and beyond is presented in **Figure** **10**. Especially, the structural/compositional evolution of direct hollow catalysts and electrocatalysts on hollow hosts is highlighted. Hollow electrocatalysts with single‐shelled, multi‐shelled, and frame‐like open features have demonstrated their advantages toward the enhancement of apparent/intrinsic properties. Heterostructured electrocatalysts based on hollow hosts have shown their superiority with long‐term stability for water splitting. The typical examples of the hollow electrocatalysts toward water splitting are summarized in **Table** **1**. Besides, we also highlight some recent examples of hollow electrocatalysts toward the hybrid water electrolysis for the generation of fuels or other value‐added products.

**Table 1:** Summary of typical examples of the hollow electrocatalysts toward water splitting

Classification	Electrocatalyst	Activity	*J* [mA cm^–2^]	*η* [mV]	Stability	Electrolyte
Single‐shelled direct electrocatalysts	Ni_2_P hollow spheres^[^ ^49^ ^]^	HER	20	130	500 CV	0.5 m H_2_SO_4_
	*β*‐Mo_2_C nanotubes^[^ ^50^ ^]^	HER	20	197	8 h	0.5 m H_2_SO_4_
			20	127	8 h	0.1 m KOH
	CoMoS_3_ hollow prisms^[^ ^51^ ^]^	HER	10	171	10 h	0.5 m H_2_SO_4_
	Ni‐Co‐MoS_2_ nanoboxes^[^ ^52^ ^]^	HER	10	155	12 h	0.5 m H_2_SO_4_
	Co_3_O_4_‐microtube arrays^[^ ^54^ ^]^	OER	150	1.59 V	12 h	1.0 m KOH
	*α*‐Ni(OH)_2_ spheres^[^ ^55^ ^]^	OER	10	331	1440 min	0.1 m KOH
Multi‐shelled direct electrocatalysts	Ni‐Fe LDH hollow nanoprisms^[^ ^56^ ^]^	OER	10	280	1000 cycles	1.0 m KOH
	Ni‐Fe LDH double‐shelled nanocages^[^ ^60^ ^]^	OER	20	246	50 h	1.0 m KOH
	CoP HoMSs with close duplicated shells^[^ ^27^ ^]^	HER	10	93	36 000 s	1.0 m KOH
		OER	10	294	36 000 s	
		OWS	10	1.57 V	100 000 s	
Frame‐like direct electrocatalysts	Pt_3_Ni frames/Ni(OH)_2_/C^[^ ^40^ ^]^	HER	12.579	100	10 000 CV	0.1 m KOH
	IrNiCu double‐layered nanoframe^[^ ^62^ ^]^	OER	10	302 ± 7	2500 CV	0.1 m HClO_4_
	Ni‐Co mixed oxide cages^[^ ^68^ ^]^	OER	10	380	10 h	1.0 m KOH
	Co‐Fe oxide frame‐like superstructures^[^ ^67^ ^]^	OER	10	340	8.6 h	1.0 m KOH
	Co_3_O_4_‐Mo_2_N nanoframes^[^ ^69^ ^]^	HER	10	100	–	1.0 m KOH
		OER	10	220	20 h	
Heterostructured electrocatalysts	Ni‐Fe‐LDH/CNT^[^ ^72^ ^]^	OER	10	1.538 V	1000 s	0.1 m KOH
				1.477 V	1000 s	1.0 m KOH
	N‐doped carbon nanotube frameworks^[^ ^73^ ^]^	OER	10	1.60 V	10 000 s	1.0 m KOH
	Ni‐N_4_/GHSs/Fe‐N_4_^[^ ^74^ ^]^	OER	10	390	10 000 s	0.1 m KOH
	Ni‐doped FeP/carbon hollow nanorods^[^ ^76^ ^]^	HER	10	72	12 h	0.5 m H_2_SO_4_
				117	12 h	1.0 m PBS
				95	12 h	1.0 m KOH
	Fe(OH)*_x_* @Cu‐MOF nanoboxes^[^ ^77^ ^]^	HER	10	112	30 h	1.0 m KOH

Despite great advances have been achieved, investigations of hollow structured electrocatalysts for water splitting are still at the proof‐of‐concept level. Based on the research trends of hollow catalysts, herein, we propose several directions to be considered in the near future for new discoveries in this field. 1) In order to meet the actual demand for the industry, economical and scalable synthetic methods should be further developed to prepare hollow electrocatalysts with high activity and durability. 2) From the aspect of structural design, in‐depth understandings of the basic material science and chemical reaction mechanism are still required to identify the complex effects (strain effect, ensemble effect, synergistic effect, etc.) of hollow structures on the underlying origins for electrochemical water splitting. 3) The role of temporal–spatial ordering in mass transfer, storage, and release in the hollow structured electrocatalysts should be further investigated based on theoretical calculations and experimental data to optimize these unique nanoreactors. 4) From the performance point of view, the in situ/operando high‐resolution characterizations are needed to study the reason of performance degradation for practical utilization of hollow electrocatalysts in electrolyzed water equipment. 5) For innovative strategies for nonconventional water splitting, precise structural and compositional controls are highly demanded to alter the surface chemistry of hollow electrocatalysts for target reactions beyond OER.

Given the rapid progress and continuous research activities in this field, we are optimistic that the future design of hollow nanostructures can solve the above challenges to promote their commercial applications.

## Conflict of Interest

The authors declare no conflict of interest.
